# Merkel cell polyomavirus and *trichodysplasia spinulosa-*associated polyomavirus DNAs and antibodies in blood among the elderly

**DOI:** 10.1186/1471-2334-12-383

**Published:** 2012-12-28

**Authors:** Mohammadreza Sadeghi, Matti Aronen, Tingting Chen, Laura Jartti, Tuomas Jartti, Olli Ruuskanen, Maria Söderlund-Venermo, Klaus Hedman

**Affiliations:** 1 Department of Virology, Haartman Institute, University of Helsinki, Helsinki, Finland; 2 Department of Geriatrics, Turku City Hospital, Turku, Finland; 3 Department of Pediatrics, Turku University Hospital, Turku, Finland; 4 Department of Virology and Immunology, Helsinki University Central Hospital Laboratory Division, Helsinki, Finland

**Keywords:** MCPyV, TSPyV, PCR, Serology, Serum, Elderly

## Abstract

**Background:**

Merkel cell polyomavirus (MCPyV) and *trichodysplasia spinulosa-*associated polyomavirus (TSPyV) are recently found pathogens causing two rare skin disorders, Merkel cell carcinoma (MCC) and trichodysplasia spinulosa (TS). MCC is proportionally common in the elderly and most often is associated with immunosuppression. TS is a folliculocentric infection seen in patients in an immunocompromised state. Little or no baseline information exists, however, on the prevalences of these two viruses among the elderly. Epidemiologic data on this population could help in understanding their natural biology. We wished to determine the occurrences and blood levels of MCPyV and TSPyV DNAs among the elderly and any association between the prevalences of their corresponding antiviral IgG antibodies.

**Methods:**

From 394 hospitalized elderly individuals (age ≥65 years) with respiratory symptoms, cardiovascular, and other diseases, we studied 621 serum samples by four different real-time quantitative (q) PCRs, two for the DNAs of MCPyV and two for TSPyV. The IgG antibodies for both viruses among 481 serum samples of 326 subjects were measured with enzyme immunoassays (EIAs), using as antigen recombinant virus-like particles (VLPs).

**Results:**

Of the 394 patients, 39 (9.9%) were positive at least once for MCPyV DNA by the LT PCR, and 33 (8.4%) by the VP1 PCR, while 6 (1.5%) were positive by both PCR assays. In general, the viral DNA copy numbers were low. In sharp contrast, no TSPyV DNA was detectable with qPCRs for the corresponding genomic regions. The IgG seroprevalence of MCPyV was 59.6% and of TSPyV, 67.3%.

**Conclusions:**

MCPyV DNA, unlike TSPyV DNA, occurs in low copy number in serum samples from a notable proportion of aging individuals. Whether this reflects enhanced viral replication possibly due to waning immune surveillance, and is associated with increased MCC risk, deserves exploration.

## Background

Merkel cell polyomavirus (MCPyV) causes Merkel cell carcinoma (MCC) [1], an aggressive skin cancer that is highly unusual before age 50 [2,3]. The elderly, immunosuppressed individuals and post-transplant patients, and Caucasians exposed to excessive UV light, are at increased risk [4,5]. MCPyV infection, defined by serology or detection of viral DNA, is prevalent [6-10]. The presence of MCPyV DNA at high copy number and tumor-specific mutations in MCPyV genomes appear in tumor tissue but not in healthy tissue [11]. Interestingly, similar truncating mutations have been described in chronic lymphocytic leukemia (CLL) [12].

Ubiquitous presence of MCPyV DNA has become apparent in cutaneous swabs from clinically healthy subjects at prevalences of 40 to 60% [13,14]. Besides in skin, viral DNA has been detected at lower frequencies also in respiratory secretions, on the oral and anogenital mucosa, and in the digestive tract [9]. Furthermore, by examining fetal autopsy samples, we obtained data to rule out MCPyV vertical transmission [15]. Therefore, the exact mode of transmission remains to be elucidated and could involve cutaneous, fecal-oral, mucosal, or respiratory routes. Moreover, the presence of the MCPyV genome has also been reported in peripheral blood mononuclear cells (PBMC) from adult HIV/AIDS patients without MCC and from healthy blood donors at low DNA copy numbers [16,17]. Among healthy subjects, MCPyV exposure as measured by serum antibodies to viral capsid proteins appears to be wide [18,19]. Tolstov et al. showed seroprevalences of 43% among children aged 2 to 5 years, and 80% among adults older than 50 [18]. We and others also observed frequent primary exposure to MCPyV during childhood and a trend toward increasing seroprevalence among adults [20,21].

Trichodysplasia spinulosa-associated polyomavirus (TSPyV)*,* the eighth human polyomavirus, was detected by rolling circle amplification (RCA) after the identification of human polyomaviruses 6 and 7 in 2010 [14,22]. Trichodysplasia spinulosa (TS) is a rare, disfiguring skin condition that affects immunocompromised solid organ transplant patients and lymphocytic leukemia patients, universally involving the central face [23-25]. Its discoverers further showed the presence of TSPyV DNA in eyebrow hairs of 4% of 69 renal transplant patients without TS and a lack of TSPyV DNA in human pilomatricomas [22,26]. High prevalence (100%) and load (∼10^6^ copies/cell), of TSPyV DNA in TS lesions, and abundant expression of TSPyV VP1 in the affected hair follicle cells evidenced that active TSPyV infection is associated with TS and apparently essential in its pathogenesis [27]. Two recent serology studies showed that TSPyV circulates widely in the human population (prevalences of 10% in small children to 80% in adults), and primary exposure is extensive in childhood, beginning at age 1 or 2 years [28,29]. TSPyV positivity of nasopharyngeal and fecal samples from an immunosuppressed child (heart transplant recipient) without TS suggests respiratory or fecal–oral route of transmission [30].

Data regarding MCPyV, TSPyV and aging are scarce. Additional epidemiological deta on elderly persons, regarding serum antibody responses and genome prevalence is needed. To our knowledge the present collection of sera is the first sizeable material that has been studied for the presence of MCPyV and TSPyV in aging individuals in order to determine whether and to what extent these viruses appear in this population at elevated risk of MCC. We studied a large number of serum samples from aging (≥65 years) representatives of the general population by real-time quantitative (q) PCRs for the DNAs of MCPyV and TSPyV by using primer sets directed against the genes encoding large-T antigen 1 (LT1) and viral protein 1 (VP1). In addition, the IgG antibodies for the two viruses were measured with EIAs by using as an antigen the corresponding VP1 virus-like particles (VLPs).

## Methods

### Study populations

For determination of MC and TS polyomavirus DNAs and IgG antibody seroprevalences, 621 blood samples were collected from 394 hospitalized senior citizens with respiratory symptoms or suspected pneumonia, cardiovascular, and other diseases in the city hospital of Turku, Finland, between July 2007 and April 2009. The criteria for sampling were age 65 years or older, disease requiring hospitalization, and a written assignment from the patient or trustee. Patients who came for a short elective operation were excluded from the study. The study protocol was approved by the Ethics Committee of Turku University Hospital.

### Sample collection

Eligible patients were informed of this study at hospital entry. After signing the consent, the patients or trustees were interviewed, and hospital records reviewed for clinical history. Nasopharyngeal swab samples (flocked swab, 520CS01, Copan, Brescia, Italy) and serum samples were collected at hospital entry and after two weeks or at discharge for detection of acute infections. The swabs in dry tubes and serum samples were stored at -80°C. Disposable gloves were used to prevent contamination.

### DNA extraction

The DNA Mini kit (Qiagen, Crawley, UK) was used according to the manufacturer's instructions for nucleic-acid extraction. A negative control of molecular biology-grade water was extracted and included in the PCR between sets of 10 samples. MCPyV DNA is known to occur on virtually all environmental surfaces that have been in contact with human skin [31]. During sample processing, in addition to routine PCR precautions, we always wore double disposable gloves and frequently changed them as well as avoided touching anything except pipettes and used aliquoted reagents.

### Real-time PCR assay for detection of MCPyV and TSPyV

Two published primer sets targeting conserved sequences in the MCPyV genome, the large T antigen (LT) gene, and the viral capsid protein (VP1) gene (Table 1) were used according to Goh et al [32]. PCR was done with the ABI PRISM 7700 Sequence Detector (Applied Biosystems) thermal cycler using the TaqMan universal PCR master mix (PE Applied Biosystems), and the settings were 52°C for 2 min, 95°C for 10 min, followed by 45 cycles of 95°C for 10 s and 60°C (LT assay) or 58°C (VP1 assay) for 1 min. For both assays, control plasmids were cloned from amplicons of PCR-positive tonsillar samples [33] by means of the CloneJET™ PCR Cloning Kit (Thermo Scientific). Serial dilutions of the plasmids allowed determination of assay sensitivity. In each assay, five copies per reaction were reproducibly positive, corresponding to 200 copies/mL of serum. For contamination control, in addition to DNA extraction controls, we included 30 controls of molecular biology-grade water per run of 54 DNA extractions. The MCPyV qPCR products were purified for automated sequencing with the High Pure PCR product purification kit (Roche). The resulting DNA sequences were aligned by means of the Basic Local Alignment Search Tool (BLAST) against the MCPyV sequences in GenBank.

**Table 1:** Primers and probes used to detect TSPyV and MCPyV

**Virus**	**Primers & probes sequence (5**^′^-->**3**^′^**)**	**Region**	**Amplicon size**
MCPyV	FWD-CCACAGCCAGAGCTCTTCCT	LT	140
	REV-TGGTGGTCTCCTCTCTGCTACTG		
	**FAM-TCCTTCTCAGCGTCCCAGGCTTCA-TAMRA**		
MCPyV	FWD-TGCCTCCCACATCTGCAAT	VP1	59
	REV-GTGTCTCTGCCAATGCTAAATGA		
	**6FAM-TGTCACAGGTAATATC-MGBNFQ**		
TSPyV	FWD-TGTGTTTGGAAACCAGAATCATTTG	LT	140
	REV-TGCTACCTTGCTATTAAATGTGGAG		
	**FAM-TTCTTCTTCCTCCTCATCCTCCACCTCAAT-BHQ1**		
TSPyV	FWD-AGTCTAAGGACAACTATGGTTACAG	VP1	140
	REV-ATTACAGGTTAGGTCCTCATTCAAC		
	**FAM-ACAGCAGTGACCAGGACAAGCCTACTTCTG-BHQ1**		
Tail Sequence	AACTGACTAAACTAGGTGCCACGTCGTGAAAGTCTGACAAGTGTCTCTGCCAA TGCTAAATGA		

As the MCPyV VP1 PCR product was too short for direct sequencing, we added a 40 base pairs (bp) nonspecific nucleotide tail [34] (Table 1) in addition to a poly(C) to the 5^′^ end of the sequencing primers to accomplish a product of 110 bp. The neutral sequence is a randomly generated sequence not matching any sequence in a BLAST search.

For detection of TSPyV, we applied published qPCRs, with primer pairs targeting the VP1 and LT genes [22] but changed the quenching dye TAMRA to BHQ1 on the 3^′^ base of the probes (Table 1). While annealing was done for 15 s at 62°C, the cycling conditions were otherwise identical to those of the MCPyV protocol. For use as positive controls and to determine assay sensitivities due to the lack of known positive samples, the TSPyV LT and VP regions were synthesized and cloned into pUC57 by GenScript (Piscataway, NJ, USA). The detection limit of each assay was 5 target copies per reaction, corresponding to 200 copies/mL of serum.

### MCPyV and TSPyV serology

MCPyV and TSPyV IgG antibodies in 481 serum samples from 326 subjects (available from the initial 621 samples from 394 subjects) were measured by in-house enzyme immunoassays (EIA) based on virus protein 1 (VP1) virus-like particles (VLPs) showing no antigenic cross-reactivity between the two viruses [21,29]. Briefly, recombinant baculovirus genomes containing the MCPyV and TSPyV VP1 gene sequence were generated by using the Bac-to-Bac expression system in *S. frugiperda* (*Sf*) 9 insect cells. The VLPs were biotinylated and used as antigens in an indirect EIA assay, as described [21,29]. The cut off values defining a positive IgG result were 0.150 and 0.240 OD units at 492 nm for MCPyV and TSPyV, respectively [21,29].

## Results

### Patient characteristics

The median age of the 394 patients was 82 years (range 65 to 100), and from those 226 (57%) were female. Sera tested in this study were from three groups of patients; Group I consisted of 111 serum samples obtained from patients with respiratory disease (chronic obstructive pulmonary disease, asthma, other lung disease). Group II included 209 serum samples from patients with cardiovascular disease (stroke, coronary artery disease, myocardial infarction, heart failure, heart dysrhythmia). Group III included 301 samples from patients with other diseases (hypertension, cancer, depression, dementia, rheumatic disease) (Table 2).

**Table 2:** Associations between patient characteristics and MCPyV LT1 and VP1 DNA positivity

**Disease group**	**Sample number**	^**4**^**MCPyV VP1 odds ratio (95% confidence interval)**	^**5**^**MCPyV LT1 odds ratio (95% confidence interval)**
Respiratory disease^1^	111	**2.40 (1.13 – 5.11)**	0.630 (0.277 – 1.435)
Cardiovasevular disease^2^	209	0.57 (0.27 – 1.21)	1.348 (0.638 – 2.850)
Other disease^3^	301	0.47 (0.19 – 1.12)	1.129 (0.418 – 3.048)

Logistic regression analyses, the reference groups were those without respiratory, cardiovascular, or other diseases respectively.

^1^Respiratory disease = chronic obstructive pulmonary disease, asthma, other lung disease.

^2^Cardiovascular disease = stroke, coronary artery disease, myocardial infarction, heart failure, heart dysrhythmia.

^3^Other disease = hypertension, cancer, depression, dementia, rheumatic disease.

^4^MCPyV VP1 =Merkel cell polyomavirus viral protein 1.

^5^MCPyV LT1 =Merkel cell polyomavirus Large T Antigen1.

### MCPyV and TSPyV qPCR

MCPyV DNA detection by using qPCR for 621 serum samples of the 394 patients produced 72 positive results; 39 (9.9%) patients were positive by the LT assay, 33 (8.4%) by the VP1 assay, and 6 (1.5%) by both assays (Table 3). Concerning all these numbers, a positive result had been obtained at least twice upon reproduction. The 72 patients that were PCR positive for either LT or VP had very high *C*_*t*_ values (average *C*_*t*_ values LT/VP1 = 38.67/39.78), as did the six patients sera that were positive with both PCRs (average *C*_*t*_ values LT/VP1 = 37.08/37.18), indicating low viral loads. qPCR displayed *C*_*t*_ values in the 35.88 – 40 and 36.18 – 40 ranges for LT and VP assays, respectively. One-fifth of the PCR products were sequenced (13 of the LT gene and 12 of the VP1 gene). All were confirmed to be MCPyV and showed a 100% identity to each other and also to those of the previously described MCPyV strain sequences with no deletions or other mutations. The serum samples were also tested with the corresponding TSPyV qPCRs. No TSPyV DNA was emerged in any subject.

**Table 3:** Age distribution of MCPyV infected patients

**Age group (years)**	**No. of sample tested**	**MCPyV Positive patients % (VP1)**	**MCPyV Positive patients % (LT1)**
60-70	36	0.4	0.0
71-80	174	4.0	6.3
81-90	307	7.1	7.5
91-100	104	3.8	4.8
Total	621	8.4	9.9

### Associations between clinical characteristics and MCPyV DNA positivity

When we examined the association between MCPyV-DNA positivity in serum and clinical characteristics (Table 2), VP1-PCR positivity was significantly and positively associated with chronic respiratory disease (p = 0.023, Table 2). Univariable logistic regression was used to analyse the association between patient characteristics and virus etiology. Statistical significance was established at the level of P < 0.05. For statistics SAS Enterprise Guide 4.3 (SAS Institute Inc., Cary, NC, USA) was used. No associations appeared between LT1-PCR positivity and clinical condition.

### MCPyV and TSPyV serology

**Figure 1:**
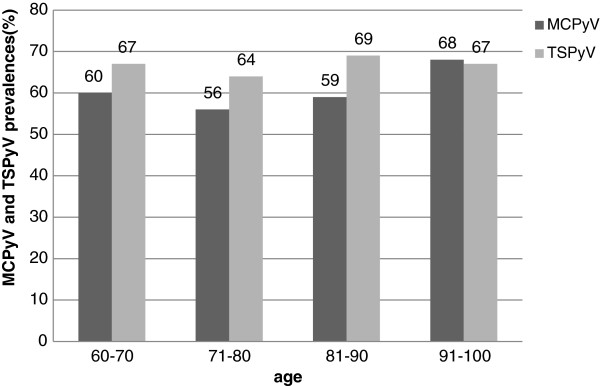
**Age-dependent prevalences of MCPyV and TSPyV antibodies.** Both MCPyV and TSPyV seroprevalences are very similar in all age groups of the elderly.

In addition, we examined by VLP-EIAs 481 corresponding serum samples from 326 elderly patients for MCPyV and TSPyV IgG antibodies. Of the 481 available sera 171 were single without follow-up sample. The IgG seroprevalence of 326 patients for MCPyV was 59.6%, and for TSPyV, 67.3%. These seroprevalences did not significantly change with increasing age (Figure 1) and we found no significant correlation between antibody levels and increasing age. Antibody titres in assays based on VLPs of MCPyV showed no correlation to the titres in similar TSPyV assays. With only two exceptions among 326 individuals, the antibody levels were almost identical in follow-up sera taken two weeks apart, ruling against recent immunogenic infections with MCPyV or TSPyV. Among the 72 MCPyV PCR positive patients 15 were MCPyV VP1 IgG negative.

### Associations between clinical characteristics and seroprevalence

Associations between seroprevalence and the diseases were calculated from clinical data available for 326 patients. The MCPyV IgG seroprevalence in the respiratory disease group was not higher than in the group without respiratory disease, OR 1.316 (0.793 – 2.182). In cardiovascular disease group the TSPyV IgG seroprecalence was lower than in a group without cardiovascular disease, OR 0.559 (0.321 – 0.973). No other associations were found. In the logistic regression analyses, the reference groups were those without respiratory, cardiovascular, or other diseases respectively.

## Discussion

Our results show the occurrence of MCPyV DNA rather commonly in sera from the elderly. The source of viral DNA in their blood is unknown. As the previously identified polyomaviruses JCV and BKV do occur at increased frequencies in blood and lymphoid tissue during host immunosuppression, and the same has been reported in some studies for the newly discovered KIPyV and WUPyV [35-37], it is tempting to speculate that with increasing age MCPyV may reactivate more often, causing viremia, especially as hematolymphoid cells may harbour MCPyV [38]. The potential in elderly individuals for MCPyV to replicate and be released into serum under special circumstances deserves further investigation.

A study of 840 serum samples for MCPyV revealed only one sample from a leukemic child to be PCR-positive; more often viral DNA was detected in tonsillar tissue of adults [33]. In a study of 635 NPA samples with exactly the same primers and probes as ours, more adults (particularly the elderly) than children were MCPyV positive [32]. Our high prevalence of MCPyV DNA among the elderly is in agreement with the findings of Goh et al. showing in NPAs MCPyV DNA more frequently among the elderly [32].

As in several other studies, viral DNA was detectable in low copy numbers, however, making the interpretation of positive results challenging [17,32,39,40]. According to others’ positivity criteria concerning VP1 and LT PCRs [32,39,40], we concluded six samples as being unequivocally positive for MCPyV. However, all 25 amplicons from the LT or VP PCRs contained the correct sequence, and the 437 negative controls (62 for DNA extraction plus 375 for qPCR) were always negative, suggesting that also the single-PCR positives were true positives. Divergent sequences among the circulating viruses could perhaps cause false negativity in the PCRs, or the low viral amounts could lead to stochastic variance in detection.

Among the 72 MCPyV PCR-positive patients 15 were MCPyV VP1 IgG negative. One possible explanation for this difference, in light of the ubiquitous presence of MCPyV DNA in superficial skin, is contamination of the needle piercing the skin during sampling. This deserves to be explored.

Of note, MCPyV detection rates by LT1, LT3, and VP1-region primers have invariably shown mutual discordance among samples of various entities [1,32,39,40]. We used primers from both the VP1 and LT regions of MCPyV and reasoned that the presence of both genes, capsid VP1 and oncogenic LT, would better indicate the presence of infectious virions, hence the stringent criterion of coupled LT and VP1 positivity for MCPyV detection by these assays. Interestingly, we found a positive association between MCPyV VP1-PCR but not LT1-PCR positivity and chronic respiratory disease. However, any conclusions about MCPyV pathogenicity in the respiratory tract cannot be drawn without epidemiologic support and further investigation with different sample types. Furthermore, it is difficult to resolve whether the higher prevalence by the VP1 assay was due to increased sensitivity or to a difference in genome identity sequences among the MCPyV strains.

The prevalence of MCPyV antibody positivity increases with age throughout life [18-21,41,42]. Our MCPyV serology results also showed that the majority of the elderly have been exposed to MCPyV with similar seroprevalences in all subgroups.

Taken together, MCPyV DNA appeared in serum in low copy numbers in many aging individuals. Serological results have shown that MCPyV infection is common, but only rarely leads to MCC. The presence of the virus alone is insufficient for tumor development. While MCC tumors require specific mutations (both T antigen truncation and genomic integration) [1], and in most cases immunosuppression, additional risk factors and viral changes are required before clinically apparent MCC emerges.

TSPyV is a ubiquitous virus that frequently infects the general population. To determine the exposure history and activity of infection among the aging, we conducted a survey by molecular and serologic tests. In contrast to MCPyV, no TSPyV DNA appeared in the elderly subjects’ sera. For one explanation of these negative PCR findings, TSPyV viremia may be of short duration. Whether TSPyV infections are able to persist is unknown, but likely, based on results for other polyomavirus infections. Our TSPyV IgG data confirmed two recent serologic reports showing TSPyV circulation in the general population [28,29]. The seroprevalence we found for TSPyV among the elderly was high (>60%) with no variation according to advancing age, and comparable with that for MCPyV.

## Conclusions

These results indicate that MCPyV DNA, unlike TSPyV DNA, occurs in low copy numbers in serum in a notable proportion of aging individuals. Whether the enhanced viral replication in our elderly participants is a reflection of waning immune surveillance and is correlated with increased MCC risk deserves exploration. Antibodies against MCPyV and TSPyV occurred at high rates in serum samples from the elderly.

## Competing interests

The authors declare that they have no competing interests.

## Authors’ contributions

MS carried out the molecular and serological studies and drafted the manuscript. MA, LJ, TJ, and OR provided the study materials. TC produced the recombinant VLPS and participated in the serological study. MS-V and KH designed, coordinated, and participated in writing the manuscript. All authors read, revised, and approved the final version of the manuscript.

## Pre-publication history

The pre-publication history for this paper can be accessed here:

http://www.biomedcentral.com/1471-2334/12/383/prepub

## References

[B1] Feng H Shuda M Chang Y Moore PS Clonal integration of a polyomavirus in human Merkel cell carcinoma Science 2008 319 5866 1096 1100 10.1126/science.1152586 18202256 PMC2740911

[B2] Agelli M Clegg LX Epidemiology of primary Merkel cell carcinoma in the United States J Am Acad Dermatol 2003 49 5 832 841 10.1016/S0190-9622(03)02108-X 14576661

[B3] Kukko H Bohling T Koljonen V Tukiainen E Haglund C Pokhrel A Sankila R Pukkala E Merkel cell carcinoma - A population-based epidemiological study in Finland with a clinical series of 181 cases Eur J Cancer 2011 48 5 737 42 21729823 10.1016/j.ejca.2011.06.001

[B4] Majewska H Biernat W Merkel cell carcinoma. Pathological and molecular aspects of diagnosis and clinical features Pol J Pathol 2010 61 3 117 123 21225493

[B5] Schrama D Becker JC Merkel cell carcinoma - pathogenesis, clinical aspects and treatment J Eur Acad Dermatol Venereol 2011 25 10 1121 1129 10.1111/j.1468-3083.2011.04032.x 21923810

[B6] Dalianis T Ramqvist T Andreasson K Kean JM Garcea RL KI, WU and Merkel cell polyomaviruses: a new era for human polyomavirus research Semin Cancer Biol 2009 19 4 270 275 10.1016/j.semcancer.2009.04.001 19416753

[B7] Gandhi RK Rosenberg AS Somach SC Merkel cell polyomavirus: an update J Cutan Pathol 2009 36 12 1327 1329 10.1111/j.1600-0560.2009.01464.x 19878388

[B8] Gjoerup O Chang Y Update on human polyomaviruses and cancer Adv Cancer Res 2010 106 1 51 20399955 10.1016/S0065-230X(10)06001-X

[B9] Babakir-Mina M Ciccozzi M Perno CF Ciotti M The novel KI, WU, MC polyomaviruses: possible human pathogens? New Microbiol 2011 34 1 1 8 21344140

[B10] Moens U Ludvigsen M Van Ghelue M Human polyomaviruses in skin diseases Patholog Res Int 2011 2011 123491 21941687 10.4061/2011/123491 PMC3173887

[B11] Shuda M Feng H Kwun HJ Rosen ST Gjoerup O Moore PS Chang Y T antigen mutations are a human tumor-specific signature for Merkel cell polyomavirus Proc Natl Acad Sci U S A 2008 105 42 16272 16277 10.1073/pnas.0806526105 18812503 PMC2551627

[B12] Pantulu ND Pallasch CP Kurz AK Kassem A Frenzel L Sodenkamp S Kvasnicka HM Wendtner CM Zur Hausen A Detection of a novel truncating Merkel cell polyomavirus large T antigen deletion in chronic lymphocytic leukemia cells Blood 2010 116 24 5280 5284 10.1182/blood-2010-02-269829 20817850

[B13] Foulongne V Kluger N Dereure O Mercier G Moles JP Guillot B Segondy M Merkel cell polyomavirus in cutaneous swabs Emerg Infect Dis 2010 16 4 685 687 10.3201/eid1604.091278 20350388 PMC3321950

[B14] Schowalter RM Pastrana DV Pumphrey KA Moyer AL Buck CB Merkel cell polyomavirus and two previously unknown polyomaviruses are chronically shed from human skin Cell Host Microbe 2010 7 6 509 515 10.1016/j.chom.2010.05.006 20542254 PMC2919322

[B15] Sadeghi M Riipinen A Vaisanen E Chen T Kantola K Surcel HM Karikoski R Taskinen H Soderlund-Venermo M Hedman K Newly discovered KI, WU, and Merkel cell polyomaviruses: no evidence of mother-to-fetus transmission Virol J 2010 7 251 10.1186/1743-422X-7-251 20860804 PMC2955715

[B16] Shuda M Arora R Kwun HJ Feng H Sarid R Fernandez-Figueras MT Tolstov Y Gjoerup O Mansukhani MM Swerdlow SH Chaudhary PM Kirkwood JM Nalesnik MA Kant JA Weiss LM Moore PS Chang Y Human Merkel cell polyomavirus infection I. MCV T antigen expression in Merkel cell carcinoma, lymphoid tissues and lymphoid tumors Int J Cancer 2009 125 6 1243 1249 10.1002/ijc.24510 19499546 PMC6388400

[B17] Pancaldi C Corazzari V Maniero S Mazzoni E Comar M Martini F Tognon M Merkel cell polyomavirus DNA sequences in the buffy coats of healthy blood donors Blood 2011 117 26 7099 101 10.1182/blood-2010-09-310557 21464370

[B18] Tolstov YL Pastrana DV Feng H Becker JC Jenkins FJ Moschos S Chang Y Buck CB Moore PS Human Merkel cell polyomavirus infection II. MCV is a common human infection that can be detected by conformational capsid epitope immunoassays Int J Cancer 2009 125 6 1250 1256 10.1002/ijc.24509 19499548 PMC2747737

[B19] Pastrana DV Tolstov YL Becker JC Moore PS Chang Y Buck CB Quantitation of human seroresponsiveness to Merkel cell polyomavirus PLoS Pathog 2009 5 9 e1000578 10.1371/journal.ppat.1000578 19750217 PMC2734180

[B20] Kean JM Rao S Wang M Garcea RL Seroepidemiology of human polyomaviruses PLoS Pathog 2009 5 3 e1000363 10.1371/journal.ppat.1000363 19325891 PMC2655709

[B21] Chen T Hedman L Mattila PS Jartti T Ruuskanen O Soderlund-Venermo M Hedman K Serological evidence of Merkel cell polyomavirus primary infections in childhood J Clin Virol 2011 50 2 125 129 10.1016/j.jcv.2010.10.015 21094082

[B22] van der Meijden E Janssens RW Lauber C Bouwes Bavinck JN Gorbalenya AE Feltkamp MC Discovery of a new human polyomavirus associated with trichodysplasia spinulosa in an immunocompromized patient PLoS Pathog 2010 6 7 e1001024 10.1371/journal.ppat.1001024 20686659 PMC2912394

[B23] Schwieger-Briel A Balma-Mena A Ngan B Dipchand A Pope E Trichodysplasia spinulosa–a rare complication in immunosuppressed patients Pediatr Dermatol 2010 27 5 509 513 10.1111/j.1525-1470.2010.01278.x 20796236

[B24] Wyatt AJ Sachs DL Shia J Delgado R Busam KJ Virus-associated trichodysplasia spinulosa Am J Surg Pathol 2005 29 2 241 246 10.1097/01.pas.0000149691.83086.dc 15644782

[B25] Tan BH Busam KJ Virus-associated Trichodysplasia spinulosa Adv Anat Pathol 2011 18 6 450 453 10.1097/PAP.0b013e318234aad2 21993271

[B26] Kanitakis J Kazem S Van Der Meijden E Feltkamp M Absence of the trichodysplasia spinulosa-associated polyomavirus in human pilomatricomas Eur J Dermatol 2011 21 3 453 454 21680290 10.1684/ejd.2011.1395

[B27] Kazem S van der Meijden E Kooijman S Rosenberg AS Hughey LC Browning JC Sadler G Busam K Pope E Benoit T Fleckman P de Vries E Eekhof JA Feltkamp MC Trichodysplasia spinulosa is characterized by active polyomavirus infection J Clin Virol 2012 53 3 225 230 10.1016/j.jcv.2011.11.007 22196870

[B28] van der Meijden E Kazem S Burgers MM Janssens R Bouwes Bavinck JN de Melker H Feltkamp MC Seroprevalence of trichodysplasia spinulosa-associated polyomavirus Emerg Infect Dis 2011 17 8 1355 1363 21801610 10.3201/eid1708.110114 PMC3381547

[B29] Chen T Mattila PS Jartti T Ruuskanen O Soderlund-Venermo M Hedman K Seroepidemiology of the Newly Found Trichodysplasia Spinulosa-Associated Polyomavirus J Infect Dis 2011 204 10 1523 6 10.1093/infdis/jir614 21926381

[B30] Siebrasse EA Bauer I Holtz LR Le BM Lassa-Claxton S Canter C Hmiel P Shenoy S Sweet S Turmelle Y Shepherd R Wang D Human polyomaviruses in children undergoing transplantation, United States, 2008-2010 Emerg Infect Dis 2012 18 10 1676 1679 10.3201/eid1810.120359 23017293 PMC3471627

[B31] Foulongne V Courgnaud V Champeau W Segondy M Detection of Merkel cell polyomavirus on environmental surfaces J Med Virol 2011 83 8 1435 1439 10.1002/jmv.22110 21618553

[B32] Goh S Lindau C Tiveljung-Lindell A Allander T Merkel cell polyomavirus in respiratory tract secretions Emerg Infect Dis 2009 15 3 489 491 10.3201/eid1503.081206 19239773 PMC2681127

[B33] Kantola K Sadeghi M Lahtinen A Koskenvuo M Aaltonen LM Mottonen M Rahiala J Saarinen-Pihkala U Riikonen P Jartti T Ruuskanen O Soderlund-Venermo M Hedman K Merkel cell polyomavirus DNA in tumor-free tonsillar tissues and upper respiratory tract samples: implications for respiratory transmission and latency J Clin Virol 2009 45 4 292 295 10.1016/j.jcv.2009.04.008 19464943 PMC7172143

[B34] Binladen J Gilbert MT Campos PF Willerslev E 5′-tailed sequencing primers improve sequencing quality of PCR products Biotechniques 2007 42 2 174 176 10.2144/000112316 17373481

[B35] Allander T Andreasson K Gupta S Bjerkner A Bogdanovic G Persson MA Dalianis T Ramqvist T Andersson B Identification of a third human polyomavirus J Virol 2007 81 8 4130 4136 10.1128/JVI.00028-07 17287263 PMC1866148

[B36] Gaynor AM Nissen MD Whiley DM Mackay IM Lambert SB Wu G Brennan DC Storch GA Sloots TP Wang D Identification of a novel polyomavirus from patients with acute respiratory tract infections PLoS Pathog 2007 3 5 e64 10.1371/journal.ppat.0030064 17480120 PMC1864993

[B37] Sharp CP Norja P Anthony I Bell JE Simmonds P Reactivation and mutation of newly discovered WU, KI, and Merkel cell carcinoma polyomaviruses in immunosuppressed individuals J Infect Dis 2009 199 3 398 404 10.1086/596062 19090778

[B38] Mertz KD Junt T Schmid M Pfaltz M Kempf W Inflammatory monocytes are a reservoir for Merkel cell polyomavirus J Invest Dermatol 2010 130 4 1146 1151 10.1038/jid.2009.392 20016500

[B39] Bialasiewicz S Lambert SB Whiley DM Nissen MD Sloots TP Merkel cell polyomavirus DNA in respiratory specimens from children and adults Emerg Infect Dis 2009 15 3 492 494 10.3201/eid1503.081067 19239774 PMC2681122

[B40] Gustafsson B Honkaniemi E Goh S Giraud G Forestier E von Dobeln U Allander T Dalianis T Bogdanovic G KI, WU, and Merkel Cell Polyomavirus DNA was not Detected in Guthrie Cards of Children who Later Developed Acute Lymphoblastic Leukemia J Pediatr Hematol Oncol 2012 34 5 364 367 22713707 10.1097/MPH.0b013e318241fb52

[B41] Carter JJ Paulson KG Wipf GC Miranda D Madeleine MM Johnson LG Lemos BD Lee S Warcola AH Iyer JG Nghiem P Galloway DA Association of Merkel cell polyomavirus-specific antibodies with Merkel cell carcinoma J Natl Cancer Inst 2009 101 21 1510 1522 10.1093/jnci/djp332 19776382 PMC2773184

[B42] Touze A Gaitan J Arnold F Cazal R Fleury MJ Combelas N Sizaret PY Guyetant S Maruani A Baay M Tognon M Coursaget P Generation of Merkel cell polyomavirus (MCV)-like particles and their application to detection of MCV antibodies J Clin Microbiol 2010 48 5 1767 1770 10.1128/JCM.01691-09 20181914 PMC2863896

